# Experimental and In Silico Studies on the Development of an Electrochemical Biosensor for the Quantification of H_2_O_2_ Based on the ChOx Enzyme

**DOI:** 10.3390/bios15050279

**Published:** 2025-04-29

**Authors:** Elvis Ortiz-Santos, Gabriela Valdés-Ramírez, Cesar Millán-Pacheco, Iris N. Serratos, Maria Luisa Lozano-Camargo, Pablo Dalmasso, Gustavo A. Rivas, Laura Galicia

**Affiliations:** 1 Departamento de Química, Universidad Autónoma Metropolitana Unidad Iztapalapa (UAM-I), Av. San Rafael Atlixco 186, Leyes de Reforma 1ra Secc., Mexico City 09340, Mexico; gabrielavr@xanum.uam.mx (G.V.-R.); insa@xanum.uam.mx (I.N.S.); lgl@xanum.uam.mx (L.G.); 2 Facultad de Farmacia, Universidad Autónoma del Estado de Morelos (UAEM), Cuernavaca 62209, Mexico; cmp@uaem.mx; 3 Departamento de Ingeniería, Tecnológico de Estudios Superiores del Estado de México (TESOEM), La Paz 56400, Mexico; maria.lozano@tesoem.edu.mx; 4 Centro de Investigación en Química Aplicada (CIQA), CONICET, Departamento de Ingeniería Química, Facultad Regional de Córdoba, Universidad Nacional Tecnológica (UTN), Maestro López esq. Cruz Roja, Córdoba 5016, Argentina; pdalmasso@frc.utn.edu.ar; 5 Instituto de Investigaciones en Físico-Química de Córdoba (INFIQC), Universidad Nacional de Córdoba (UNC), Av. Haya de la Torre, Córdoba 5000, Argentina; grivas@fcq.unc.edu.ar

**Keywords:** cholesterol oxidase, hydrogen peroxide, sensing platform, molecular recognition, in silico studies

## Abstract

This work presents the development of a biosensing platform for hydrogen peroxide (H_2_O_2_) electrochemical reduction. The developed platform uses a multi-walled carbon nanotube paste (PMWCNT) and the enzyme cholesterol oxidase (ChOx). The supramolecular architecture of the PMWCNT/ChOx platform was characterized using cyclic voltammetry, electrochemical impedance spectroscopy, and amperometry. The results indicated that the presence of ChOx enhances the sensitivity of electrochemical detection for H_2_O_2_ by 21 times compared to that without ChOx. The designed electrochemical sensing bio-platform for H_2_O_2_ shows a sensitivity of 26.15 µA/mM in the linear range from 0.4 to 4.0 mM, an LOD of 0.43 µM, and an LOQ of 1.31 µM. Furthermore, in silico studies (molecular dynamics simulations, molecular docking assays, and binding free energy calculations (Δ*G*_b_)) were carried out to characterize and validate the molecular interaction between ChOx and H_2_O_2_. The computed data confirmed that the binding is spontaneous, and the type of labile interaction promotes a rapid electrochemical reduction of H_2_O_2_.

## 1. Introduction

Hydrogen peroxide (H_2_O_2_) is the most common and stable type of reactive oxygen species (ROS) [1,2]. It is produced as a by-product of biochemical reactions, including several enzymatic reactions, mitochondrial respiration, and metabolic processes in living organisms [3,4,5]. However, elevated levels of H_2_O_2_ in the human body can lead to protein and DNA damage; it is also associated with some pathological conditions such as asthma, atherogenesis, cancer, Parkinson’s, and Alzheimer’s diseases, and cardiovascular dysfunctions [5]. Additionally, H_2_O_2_ is produced and detected in different fields, including environmental science, chemistry, food processing, textile, pharmaceutical, and biomedicine, where it can be used either as a reducing or as an oxidizing agent [6,7,8,9,10].

Due to the significance of H_2_O_2_ in various fields, its determination in aqueous and non-aqueous samples is commonly conducted using analytical techniques as colorimetry, chemiluminescence, UV-vis spectroscopy, fluorescence, high-performance liquid chromatography (HPLC), and electroanalysis [11,12,13,14,15,16,17,18,19,20]. Among these techniques, electrochemical sensors for H_2_O_2_ represent an interesting alternative regarding cost, time efficiency, sample pre-treatment, sensitivity, and selectivity. When designing and constructing these electrochemical sensors, various materials are utilized. There are two main types of electrochemical sensors: the non-enzymatic and the enzymatic sensors. On the one hand, the former has utilized nanomaterials such as carbon nanotubes (CNTs), graphene, carbon dots, metal–organic frameworks (MOFs), or metal nanoparticles [21,22,23,24,25,26,27,28]. Additionally, the use of redox mediators combined with enzymes like horseradish peroxidase (HRP), catalase, or heme prosthetic groups has been the initial point for the design of H_2_O_2_ biosensors. These biosensors allow the quantification of the current changes generated by the oxidation or reduction of H_2_O_2_ [29,30,31,32,33,34,35,36]. The literature indicates that biosensors based on oxidoreductase enzymes have been intensively researched over the past decade, driven by the need for faster and more accurate protocols. Oxidoreductase enzymes are typically used as recognition agents in the development of biosensors of different analytes; in those cases, enzymes such as glucose oxidase, glutamate oxidase, lactate oxidase, uricase, pyruvate oxidase, alcohol oxidase, or cholesterol oxidase, are employed to catalyze the oxidation of the respective substrate in the presence of oxygen, generating H_2_O_2_ as a by-product. The generated H_2_O_2_ can be oxidized or reduced at the electrode surface, adjusting the produced current. The current changes can be correlated with the analyte concentration in the tested sample [37,38,39,40,41,42,43,44].

The importance of studying oxidoreductases is that they form a class of enzymes specifically involved in the catalysis of oxidation–reduction reactions. These enzymes enable the transfer of electrons from the reductant to the oxidant [45]. The enzyme cholesterol oxidase (ChOx), also known as 3β-hydroxysterol oxidase, is an alcohol flavoenzyme that functions as an oxidoreductase. This means that it contains flavin adenine dinucleotide (FAD), which allows it to catalyze the transfer of electrons between molecules, the donor CH-OH group with oxygen as acceptor [45,46]. The ChOx enzyme is known for its simplicity, specificity; it possesses remarkable stability in extreme conditions such as high temperatures and very acidic and alkaline pH values. The thermal stability of this enzyme is favorable for clinical applications. Cholesterol oxidase has gained significant attention recently due to its increasing applications in clinical practice, particularly for cholesterol determination in serum. Additionally, it plays a role in the synthesis and production of various steroids. Beyond these uses, ChOx has demonstrated strong insecticidal activity and can help regulate cholesterol levels within cells. Moreover, ChOx is implicated in the development of several diseases of bacterial origin, such as tuberculosis, as well as viral (HIV) and non-viral prion-related diseases like Alzheimer’s disease [46,47,48].

Enzymes that have FAD as a cofactor in their active site have redox properties. Currently, the enzymes employed to detect H_2_O_2_ are those that have metal ions in their active center, such as Fe (HRP, cytochrome C, cytochrome P411). This paper presents a study on a new application of the ChOx enzyme to develop an electrochemical biosensor for the electrochemical reduction of H_2_O_2_. The design features a multi-walled carbon nanotube paste electrode with immobilized Cholesterol oxidase. Experimental studies were reinforced with in silico studies based on molecular dynamics simulations and ΔG_b_^a^ energy calculations to assess the interaction between ChOx and H_2_O_2_.

## 2. Materials and Methods

### 2.1. Reagents and Instruments

Multi-walled carbon nanotubes (MWCNTs) (outer diameter: 6–13 nm, length: 2.5–20 μm, purity > 98%), mineral oil, hydrogen peroxide (H_2_O_2_, 30% *v*/*v* aqueous solution), microbial Cholesterol oxidase (ChOx) (C1235-100UN) lyophilized powder, sodium phosphate monobasic (NaH_2_PO_4_), sodium phosphate dibasic (Na_2_HPO_4_), and sulfuric acid (H_2_SO_4_) were purchased from Sigma-Aldrich (St. Louis, MO, USA). All chemicals were analytical grade and used without purification. All solutions were prepared utilizing 18.2 MΩ·cm deionized water.

Voltammetric and amperometric studies were carried out with a BAS-100 B potentiostat (Version 2.3), and a Biologic SP-150 (Version V11.42) potentiostat was utilized for electrochemical impedance spectroscopy (EIS) experiments. A bar of 5 mm diameter glassy carbon was used as the electrical contact of the working electrode assembly; a graphite cylinder was used as auxiliary electrode; and an Ag/AgCl_(sat)_ was used as reference electrode; all potentials were referred to the Ag/AgCl_(sat)_ reference electrode.

The pH measurements were carried out with a Conductronic PC45 pH meter at room temperature.

### 2.2. Software

ZView^®®^ version 3.5i was employed for EIS analysis data.

Gromacs 2019.2 is a molecular dynamics program with a wide range of applications for systems with multiple particles and energy functions. It supports multiscale techniques such as QM/MM, MM/CG, and implicit solvents [49].

Charmm-gui server is a web-based platform that provides well-established and reproducible simulation protocols for molecular simulations using various simulation potentials [50,51,52].

Adaptive Poisson–Boltzmann Solver (APBS) calculates electrostatic binding energy by solving Poisson–Boltzmann equations, incorporating both solvation and Coulombic components.

In this work, we use the Visual Molecular Dynamics (VMD) program to calculate the solvent-accessible surface area change for the non-electrostatic component of binding energy.

### 2.3. Solutions

Sodium phosphate buffer (PB) 0.050 M, pH 7.4, was used as the supporting electrolyte. PB also served as the solvent to prepare the H_2_O_2_ stock solution and the 20 U/mL ChOx enzymatic solution, both prepared daily.

### 2.4. Sensing Bioplatform Preparation

MWCNTs were activated according to [28]. Briefly, the MWCNTs were placed in 1 M nitric acid solution and sonicated for 30 min. After that, the MWCNTs were filtered and placed in 1 M sulfuric acid solution and sonicated for 30 min. This procedure was repeated twice. Finally, the activated MWCNTs were filtered and extensively washed with ethanol and acetone until the washing residues had a neutral pH.

The PMWCNT was prepared by mixing activated MWCNTs and mineral oil in a 70/30 *w*/*w* ratio.

**Scheme 1:**
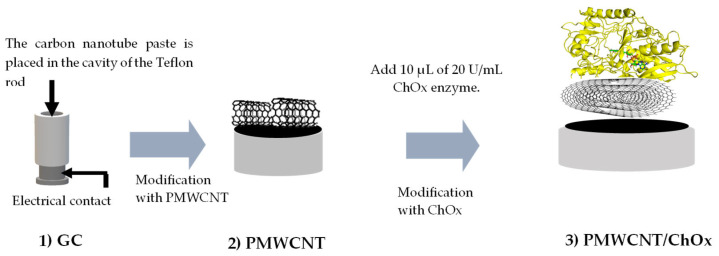
Representation of the construction of the PMWCNT/ChOx biosensing platform.

The surface of the glassy carbon cylinder was polished with 1 µm and 0.5 µm alumina; after that, it was rinsed with deionized water and sonicated for 1 min to remove any residues. Finally, the glassy carbon was rinsed with deionized water and dried with nitrogen gas, and the PMWCNT was set by placing it onto the glassy carbon (GC) contact. The surface PMWCNT/ChOx biosensing platform was prepared by drop-casting 10 μL of ChOx (20 U/mL) onto the PMWCNT. Finally, it was allowed to dry for 10 min at room temperature before being used (Scheme 1).

### 2.5. Characterization of the Electrochemical Sensing Platforms

The PMWCNT and PMWCNT/ChOx platforms were electrochemically characterized by cyclic voltammetry from −0.80 V to 0.20 V at a scan rate of 0.10 V/s in PB. Furthermore, the EIS spectrum was recorded for each sensing platform in PB. All experiments were performed at room temperature, and all potentials were referenced to the Ag/AgCl_(sat)_ electrode.

### 2.6. Electrochemical Quantification of H_2_O_2_

Each sensing platform was used as a working electrode to quantify H_2_O_2_ from 0.4 to 4.0 mM through amperometry assay by applying a constant potential in PB solution. Additionally, the analytical parameters were computed for each sensing system.

### 2.7. Molecular Dynamics Simulations of ChOx

High resolution (PDBID:1N4W) of ChOx from *Streptomyces* sp. SA-COO [53] was used for molecular simulations. 1N4W structure was processed on the Charmm-gui server [54]. ChOx was immersed in a cubic TIP3 water box with 10 Å of each protein atom to the water edge, containing 0.15 M KCl. A total of 100 ns molecular dynamics simulations by triplicate were performed using Gromacs 2019.2 [55,56,57]. Periodic boundary conditions were used. Electrostatic forces were computed with the Particle Mesh Ewald (PME) method. The Charmm36m force field was used [58,59]. The Gromacs input files were generated and utilized following the recommendations provided by the Charmm-gui server. Subsequently, clustering analysis was carried out using the gmx cluster tool within the Gromacs framework, employing the Gromos clustering method.

### 2.8. Molecular Docking

A representative structure of the molecular dynamics simulations was used to place the peroxide molecules on the presumable binding site of the ChOx. Cholesterol oxidase of *Streptomyces* sp. (PDB ID: 1MXT) was used as a reference to define the center of the molecular docking grid box [60]. The center of the grid box was located near the carboxyl group of Glu361 (20.161, −3.652, 5.814) with a size of 7.5 × 7.5 × 7.5 Å^3^. A total of 100 independent molecular docking experiments were conducted using Autodock Vina 1.2.5 [61,62]. Molecular images were realized on Maestro from Schrödinger 2023-3 [63].

### 2.9. Binding Energy Calculations Between ChOx and H_2_O_2_

The Nathan Baker’s methodology was used to determine the electrostatic components ΔG_elec_: solvation, ΔG_solv,_ and Coulombic, ΔG_Coul,_ to the free binding energy ΔG_b_ using the Adaptive Poisson–Boltzmann Solver (APBS) program [64], at pH 7.4 (considering the pH of the electrochemical experiments for the H_2_O_2_ detection).

ΔG_elec_= ΔG_solv_ + ΔG_Coul_ (1)

The non-electrostatic or non-polar component (ΔG_no-polar_) was determined by multiplying the solvent-accessible surface area change (∆ASA), calculated using the Visual Molecular Dynamics (VMD) program [65], this ∆ASA was multiplied by the surface tension of the solvent (γ = 5 Cal mol^−1^ Å^−2^), as shown in Equation (2) [66,67]:

ΔG_non-polar_ = γ (ASA_Complex_ − ASA_protein_ − ASA_ligand_) (2)

ΔG_b_ was determined as follows:

ΔG_b_ = ΔG_elec_ + ΔG_non-polar_ (3)

Therefore:

ΔG_b_^a^ = ΔG_solv_ + ΔG_Coul_ + ΔG_non-polar_ (4)

This methodology has been used in earlier reports to study mainly receptor–ligand interactions [68,69,70,71].

## 3. Results

### 3.1. Electrochemical Characterization of the Sensing Platforms

**Figure 1:**
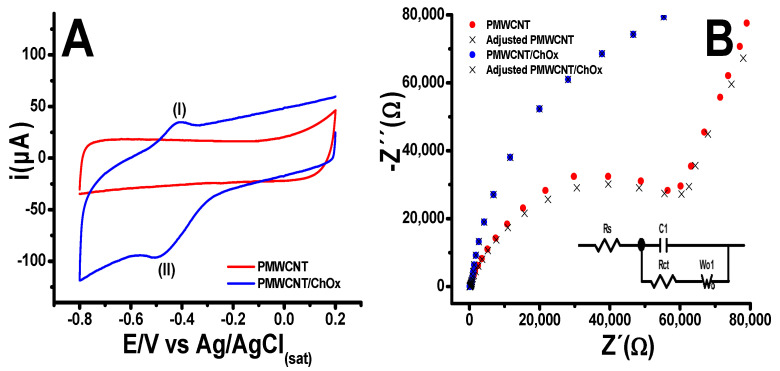
(**A**) Cyclic voltammograms for PMWCNT and PMWCNT/ChOx sensing platform in PB at 0.10 V/s scan rate. (**B**) Impedance spectra (Nyquist plots and equivalent circuit) for each sensing platform at open circuit potential in PB.

Figure 1A shows the cyclic voltammograms (CV) obtained for the PMWCNT and PMWCNT/ChOx sensing platform; the potential was swept from −0.80 to 0.20 V at a scan rate of 0.10 V/s in PB. As can be observed in the PMWCNT sensing platform, no oxidation or reduction signals were detected. However, the voltammogram for the PMWCNT/ChOx sensing platform exhibited a redox couple, showing an oxidation signal (I) at −0.38 V and a reduction (II) at −0.48 V. These signals can be assigned to the active site of the ChOx enzyme, specifically to the FAD/FADH_2_ couple. This redox couple shows a conditional potential $E_{FADH_{2}/FAD}^{0^{\prime }}=-0.43\;V.$ Similar behavior has been found for glucose oxidase immobilized on different substrates under comparable experimental conditions [72,73,74]. It is important to point out that at the PMWCNT/ChOx interface, a three-dimensional structure is formed, as has been described for other assemblies [48]. This structure enables greater interaction between the sensing platform and the solution.

The EIS experiments were conducted as a complementary method to study the electron transfer and diffusion processes at the platform/electrolyte interface. The experiments were carried out under a voltage amplitude of 0.010 V and a frequency range from 10 mHz to 200 kHz at open circuit potential (OCP) in PB solution. The Nyquist plots are shown in Figure 1B. The studied sensing platform shows a semicircle at a high-frequency range, which is related to the change transfer resistance (Rct). This is associated with the kinetics of the electron transfer of the redox indicator at the interface electrode/ dissolution. After the semicircle, a linear portion at low frequencies was observed (W_01_), which can be associated with the control diffusion ions in the PB process and the conductivity of the used materials (PMWCNT or PMWCNT/ChOx). The experimental data were adjusted to an equivalent Randles circuit, as shown in the inset of Figure 1B, where C_1_ represents the interfacial double layer capacitance, W_01_ represents the Warburg impedance, and R_s_ is the bulk solution resistance. From the equivalent circuit fitting, the R_ct_ and the C_1_ increase at the PMWCNT/ChOx, while W_01_ remains practically constant. The data for the sensing platform are summarized in Table 1.

The EIS data agree with the results obtained by cyclic voltammetry, showing that the PMWCNT/ChOx assembly displayed a higher capacitance C_1_ than the PMWCNT platform, while Rct increased by almost twice, and the W_01_ remained practically constant.

A study at different scan rates was performed; the results are shown in Figure S1A. The plot of current peak vs. scan rate (*i* vs. *v*) in Figure S1B showed that the enzyme is adsorbed onto the PMWCNT.

### 3.2. Electrochemical Detection of Hydrogen Peroxide

**Figure 2:**
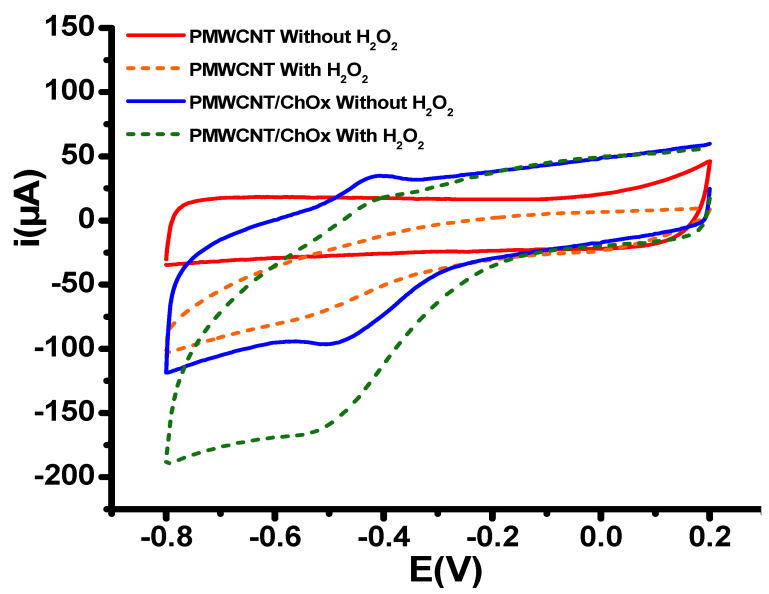
Cyclic voltammograms in PB at pH 7.4 at the potential window −0.80 to 0.2 V at a scan rate of 0.10 V/s for the PMWCNT and PMWCNT/ChOx in the absence (continuous line) and in the presence of H_2_O_2_ (dotted line).

The sensing platforms were used for the electrochemical reduction of hydrogen peroxide. Figure 2 shows the results obtained from the cyclic voltammetry of the PMWCT/ChOx platform in PB in the absence and presence of 2 mM H_2_O_2_. An experiment under similar conditions for the platform PMWCNT was conducted. It is observed that in the presence of H_2_O_2_ at the PMWCNT/ChOx platform, the reduction current increases; therefore, this is associated with the reduction of H_2_O_2_ and its interaction with the FAD/FADH_2_ of the ChOx enzyme. Although the reduction starts at −0.2 V, at −0.6 V it can be guaranteed that H_2_O_2_ is completely reduced.

### 3.3. Determination of H_2_O_2_ on the PMWCNT/ChOx

**Figure 3:**
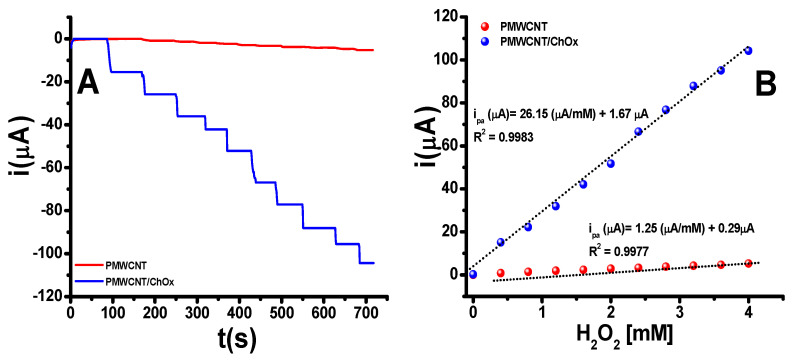
(**A**) Amperometric response for the electrochemical reduction of H_2_O_2_ at the PMWCNT and PMWCNT/ChOx sensing platforms, conducted at −0.60 V, (**B**) Calibration plots for H_2_O_2_ from 0.4 to 4.0 mM in PBS.

From the results of cyclic voltammetry studies, a constant of −0.6 V was established to ensure a complete reduction of H_2_O_2_ in the biosensing platform PMWCNT/ChOx. The H_2_O_2_ quantification was conducted in PB from 0.40 to 4.0 mM; five independent experiments were performed. Figure 3A shows a typical amperometric response for the H_2_O_2_ detection at both PMWCNT and PMWCNT/ChOx. Figure 3B presents the typical calibration plots for the H_2_O_2_ detection at PMWCNT and PMWCNT/ChOx.

As can be observed from Figure 3A,B, the presence of the ChOx enzyme at the PMWCNT/ChOx sensing bioplatform significantly increases the reduction currents compared to the PMWCNT sensing platform under the same H_2_O_2_ concentration. In both systems, linearity in the range from 0.40 to 4.0 mM was achieved. The analytical parameters, including sensitivity, low detection limit (LOD), and limit of quantification (LOQ), have been reported in Table 2. The LOD and LOQ were calculated as 3σ/m and 10σ/m, respectively [75].

The above results indicate that incorporating the ChOx enzyme into the sensing bioplatform enhances the reduction current in the electrochemical determination of H_2_O_2_. Specifically, in the PMWCNT/ChOx system, the sensitivity increases by 21 times compared to the PMWCNT sensing platform. The findings suggest that the ChOx enzyme could be a viable alternative to horseradish peroxidase (HRP), catalase, hem prosthetic groups, and/or myeloperoxidase (MPO) in the fabrication of electrochemical biosensors for H_2_O_2_ detection [27,28,29,30,31,32,33]. The increment in the reduction current at the amperometry experiments was associated with the interaction at the active site of the ChOx and the H_2_O_2_.

**Figure 4:**
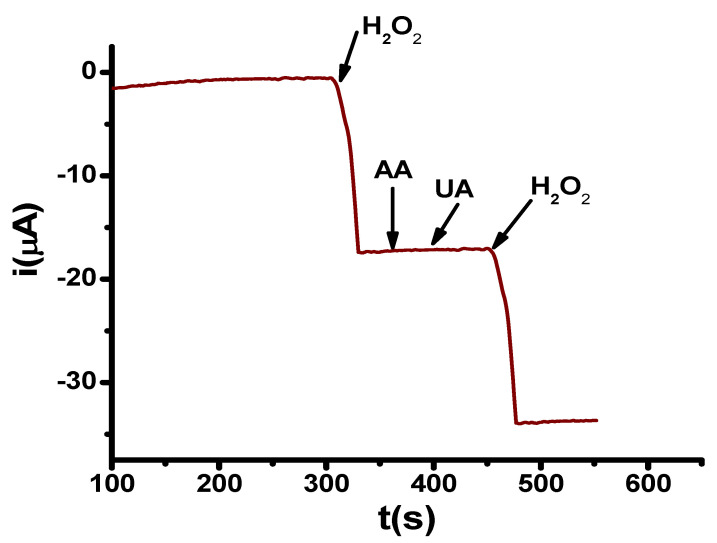
Amperometric records for additions H_2_O_2_ 0.4 mM_,_ Ascorbic acid (AA) 0.025 mM, Uric acid (UA) 0.025mM, H_2_O_2_ 0.4 mM in PB at pH 7.4 in PMWCNT/ChOx at −0.6 V.

The selectivity of the PMWCNT/ChOx was evaluated by conducting amperometric studies with additions of 0.025 mM of Ascorbic acid (AA), and 0.025 mM Uric acid (UA). For either of these, any changes in the electrochemical response were observed, as shown in Figure 4. It is important to mention that both analyzed compounds do not present a response in reduction, while they are commonly detected at oxidation, as mentioned in [76].

To better understand experimental results, the behavior of the ChOx in the sensing bioplatform and the interactions of the molecular ChOx-H_2_O_2_ complex interactions were studied by in silico studies (molecular dynamics simulations, docking assays, and binding energy calculations).

### 3.4. In Silico Studies: Interaction Between ChOx and H_2_O_2_

**Figure 5:**
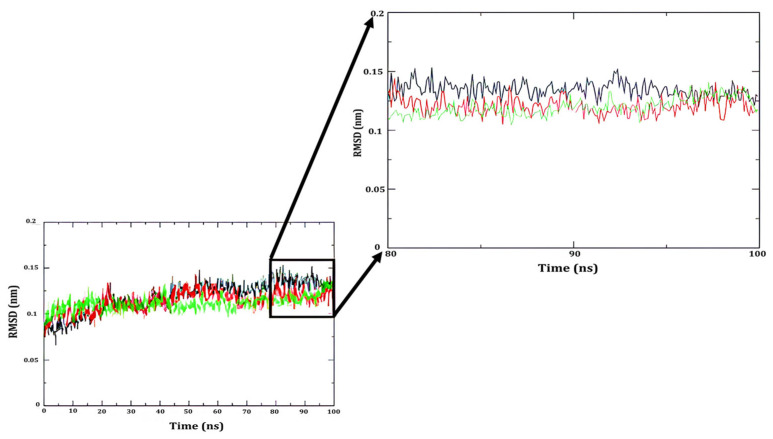
α-carbon root mean square deviation for all systems studied herein.

Three independent molecular simulations of Cholesterol oxidase from *Streptomyces* sp. *SA-COO* were realized to explore its conformational changes and to obtain a representative relaxed structure. The α-carbon root mean square deviation (RMSD) from the amino acids of the enzyme of all systems was measured for the entire simulated duration. Figure 5 shows the resulting RMSD.

As noted above, the α-carbon RMSD presented fluctuations (not higher than 1.6 Å compared to the initial structure) during the first 70 ns, as shown in Figure 5. The last 20 ns of each simulation was used for analysis (80 to 100) ns. Cluster analysis was used to obtain a representative conformation of ChOx. The structure at the center of each cluster was compared against the crystallographic structure, obtaining α-carbon RMSD of 1.32, 1.12, and 1.08 Å. Due to the low value of RMSD, one cluster structure was used to locate the predicted location of the peroxide molecule, the 1MXT structure [60], thus it was used as a template for the peroxide molecule.

**Figure 6:**
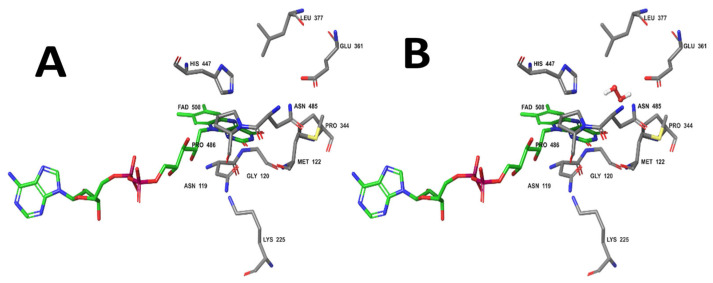
The cluster representative structure of ChOx and the probable H_2_O_2_ location are shown. (**A**) Probable residues involved in recognizing the H_2_O_2_ molecule. (**B**) H_2_O_2_ (center) and those residues at 5 Å. Molecular images were realized on Maestro from Schrödinger 2023-3 program (academic license) [64].

From the obtained in silico results, a docking study between ChOx and H_2_O_2_ was performed. The H_2_O_2_ molecule was docked on a representative structure of the enzyme obtained by molecular dynamics simulations. Figure 6A shows the active site of ChOx, which involves the Asn119, Gly120, Met122, Lys225, Pro344, Glu361, Leu377, His447, Asn485, and Pro486. According to the literature [77,78,79,80,81], the catalytic roles associated with Glu361, His447, and Asn485 have been studied extensively. Furthermore, Chen et al. [82] showed that the binding and release of O_2_ or H_2_O_2_ takes place through the hydrophobic tunnel in ChOx. Notably, the Met122, Glu361, and Asn485 are important residues because they meet the isoalloxazine ring of FAD in the tunnel in an open or closed conformation of ChOx [82].

The interaction between the cluster structure of ChOx obtained by molecular dynamics simulations and the H_2_O_2_ molecule is shown in Figure 6B. H_2_O_2_ was found in the same orientation in 94% of independent experiments with an affinity energy of −2.088 ± 0.004 kcal/mol.

The H_2_O_2_ molecule established electrostatic interactions and hydrogen bonds with Glu361, His 447, Asn 485 (Supplementary Materials Figure S2), and some hydrophobic contacts with FAD rings. These interactions are reflected in the contributions of the ΔG_b_ computing values (Table 3).

Binding energy calculations were performed, and the ΔG_b_ value from H_2_O_2_ to ChOx, as shown in Figure 6B, was negative, indicating that the reaction is spontaneous even though peroxide is a small molecule (−1.5 kJ/mol), where the binding was driven by electrostatic interactions, mainly the Coulombic component (−3.9 kJ/mol) and non-polar contacts (−3.3 kJ/mol). The solvation energy was positive because the electrostatic interactions incur a penalty for the desolvation of individual molecules [83,84].

According to the calculated in Equation (4) ΔG_b_^a^ = ΔG_solv_ + ΔG_Coul_ + ΔG_non-polar_, it is verified that the interaction between ChOx-H_2_O_2_ is of a labile type that allows the desorption of the product, releasing the active site to continue with a new ChOx-H_2_O_2_ interaction cycle. From the experimental data of amperometry, an increase in the peroxide reduction current is observed in the sensing bioplatform. The data help establish that this interaction is determinant in the amperometric response detected in the experimental studies, resulting in greater sensitivity for the electrochemical determination of H_2_O_2_.

## 4. Discussion

The results obtained from this research show that the presence of ChOx in the sensing platform helps enhance the global reduction current of H_2_O_2_ determination; thus, the generated biosensor functions like other classical enzymes such as HRP, catalase, or heme prosthetic groups. In this work, we have applied the electrochemical technique of cyclic voltammetryand amperometry to study the capability of ChOx to interact with H_2_O_2_, facilitating its electrochemical reduction. When we compared the catalytic activity of the two studied platforms, PMWCNT and PMWCNT/ChOx, we found that the reduction reaction current on the PMWCNT/ChOx bio-platform was 21 times greater than that of the PMWCNT. Through electrochemical characterization using cyclic voltammetry, a redox signal was assigned to the FAD/FADH_2_ of the enzyme ChOx. Under the experimental conditions studied (phosphate buffer at pH 7.4), the conditional redox potential is $E_{FADH_{2}/FAD}^{0^{\prime }}=-0.43\;V$, similar to the values reported for this redox pair in other oxidoreductase measured under similar conditions [73,74,75].

The found behavior for the ChOx enzyme may also be similar for other enzymes where the FAD coenzyme is labile, such as in the glucose-methanol-choline (GMC) oxidoreductase class; however, if the study is conducted employing oxidoreductases where the FAD is not labile, as in the group of enzymes of the vanillyl alcohol oxidase(VAO) class, it is expected that the results will be different [45].

To understand the electrochemical response observed on the sensing bio-platform, we conducted an in silico study of the interactions with the molecular complex of ChOx. This study utilized molecular dynamics simulations, docking assays, and binding energy calculations. Through the applied methodology, we elucidated the structure of the active site of ChOx and the protein residues involved (Asn119, Gly120, Met122, Lys225, Pro344, Glu361, Leu377, His447, Asn485, Pro486, and FAD).

According to the literature [78,79,80,81,82], the catalytic roles have been associated with Glu361, His447, and Asn485, and have been studied extensively. Figure 6, presents the ChOx structure, and the residues involved in the interaction with H_2_O_2_. The applied methodology enables us to determine the interaction energy between hydrogen peroxide and the active site of ChOx, highlighting the electrostatic interactions and the formation of hydrogen bonds with Glu361, His 447, ASN 485, and hydrophobic interactions with FAD rings.

The computational study yields an interaction energy (ΔG_b_^a^ = −1.5 kJ/mol) between the ChOx and H_2_O_2_ complex.

The value of the energy obtained from the calculation is a labile type that allows for the desorption of the product, releasing the active site to continue with a new ChOx-H_2_O_2_ interaction cycle. The weak interactions between ChOx-H_2_O_2_ explain the increase in the reduction current recorded in amperometric experiments, which is associated with rapid desorption of reaction products, followed by the occupation of the active site by new hydrogen peroxide molecules to continue reacting at this catalytic site.

These results obtained from the electrochemical determination of H_2_O_2_ were compared with other measurement systems for hydrogen peroxide reduction, as shown in Table 4.

The data reported in the literature regarding the reduction of H_2_O_2_ using HRP-based biosensors combined with other materials present complex architectures. Despite some biosensor platforms showed lower detection limits and narrower linear ranges than those obtained for the sensor in this work, PMWCNT/ChOx is a viable alternative to quantify H_2_O_2_ with potential applications in textile, pharmaceutical, and food industrial samples.

## 5. Conclusions

In this work, a sensing assembly PMWCNT/ChOx, composed of multi-walled carbon nanotubes and Cholesterol oxidase, is utilized for the H_2_O_2_ electrochemical reduction. Compared to the sensing assembly without ChOx, the current signal was enhanced 21 times when comparing it with the sensing assembly of PMWCNT without ChOx, and the improvement in the current has been associated with the molecular ChOx–H_2_O_2_ complex interactions. The sensitivity value for H_2_O_2_ at the PMWCNT/ChOx platform was 26.15 ± 0.49 µA/mM, and the LOD was 0.43 µM.

The interaction between ChOx and H_2_O_2_ was investigated through in silico studies. Molecular dynamics simulations, docking assays, and binding energy calculations revealed a ΔG_b_^a^ value of −1.5 kJ/mol. This finding helps to clarify the driving forces behind the interaction of the ChOx-H_2_O_2_ complex, which enhances the reduction current of H_2_O_2_. Furthermore, our results confirm that ChOx plays an important role in the obtained amperometric response, which is observed by the increments in the reduction current and, ultimately, the improved sensitivity for H_2_O_2_ detection. The ChOx enzyme is an alternative to enzyme HRP, or heme prosthetic groups, in the construction of biosensors for the electrochemical detection of H_2_O_2_ in potential applications, the biomedical, alimentary, and environmental industries [90,91,92,93]. However, additional studies are necessary before applying the PMWCNT/ChOx biosensor for the determination and quantification of H_2_O_2_ in real samples, as these matrices may contain other reactive species that could influence the biosensor’s performance.

## Figures and Tables

**Table 1:** Equivalent circuit fitting parameters obtained for the modified electrodes.

System	R_s_ (Ω)	C_1_ (F)	R_ct_ (Ω)	W_01_(Ωs^−1/2^)
PMWCNT	144.7	2.170 × 10^−6^	126,900	42,201
PMWCNT/ChOx	143.1	1.222 × 10^−4^	245,831	40,001

**Table 2:** Analytical parameters for H_2_O_2_ electrochemical reduction at sensing platforms.

System Platform	Sensitivity (µA/mM)	LOD (µM)	LOQ (µM)
PMWCNT	1.25 ± 0.22	0.28	0.94
PMWCNT/ChOx	26.15 ± 0.49	0.43	1.31

**Table 3:** ΔG_b_ values of ChOx (cluster representative) with H_2_O_2_ determined at pH 7.0 by APBS [65] and VMD1.9.1 [66].

System	ΔG_solv_ (kJ/mol)	ΔG_Coul_ (kJ/mol)	ΔG_non-elec_ (kJ/mol)	ΔG_b_^a^ (kJ/mol)
Cluster representative structure of ChOx and its interaction with H_2_O_2_	5.7	−3.9	−3.3	−1.5

**Table 4:** Comparison of the performance of the hydrogen peroxide biosensors based on HRP.

Modified Electrode	Potential (V)	Linear Range (μM)	Detection Limit (μM)	Reference
HRP/TB/CCB	−0.25	0.429–455	0.17	[85]
HRP-MIL100(Cr)-B	−0.3	0.5–3000	0.1	[86]
HRP-BMIM-BF_4_/SWCNTs/CFUME	−0.35	0.49–10.2	0.13	[87]
HRP-MoS_2_-Gr	−0.08	0.2–1.103	0.049	[88]
Co_3_O_4_/MWCNTs/gelatin/HRP	−0.3	0.74–19	0.74	[36]
HEPNP/rGO/Au electrode	−0.45	0.01–100	0.01	[89]
HRP–AuNPs-SG/GCE	−0.60	10–1800	5	[31]
PMWCNT/ChOx	−0.60	400–4000	0.43	This work

## Data Availability

Original contributions presented in this study are included in the article/Supplementary Materials. Further inquiries can be directed to the corresponding author(s).

## Supplementary Materials

The following supporting information can be downloaded at: https://www.mdpi.com/article/10.3390/bios15050279/s1, Figure S1. (A) Cyclic voltammograms in PB at pH 7.4 in a potential window of −0.80 to 0.2 V, at different scan rates: 0.01 to 0.50 V/s. (B) Peak current vs. scan rate (*i* vs. *v*). Figure S2. Schematic representation of the interaction of the FAD cofactor and H2O2 to cluster representative structure of ChOx: (A) Interaction diagram in 2D, showing hydrogen bonds (green color, dotted line) determined by LigPlot+ program [94]. (B) Interaction map in 3D, showing hydrogen bonds (green color line) determined by PyMOL 2.4 program [95]. HIS 447 changed to HSD 447 for the proton at Nδ on its side chain by PDB2PQR web server [96].
