# Supplementary material for: Experimental and In Silico Studies on the Development of an Electrochemical Biosensor for the Quantification of H2O2 Based on the ChOx Enzyme
Source: Biosensors (Basel). 2025 Apr 29;15(5):279. doi: 10.3390/bios15050279 (PMC12109943; doi:10.3390/bios15050279)
Supplement: Supplementary file 1 [file biosensors-15-00279-s001.zip › biosensors-3503102-supplementary.pdf]

Supplementary Material

Experimental and in Silico Studies on the Development of an Electrochemical Biosensor for the Quantification of H_2O_2 Based on the ChOx Enzyme

[1] Departamento de Química, Universidad Autónoma Metropolitana Unidad Iztapalapa (UAM-I), Av. San Rafael Atlixco 186, Leyes de Reforma 1ra Secc., México City 09340, México; gabrielavr@xanum.uam.mx (G.V.-R.); insa@xanum.uam.mx (I.N.S.); lgl@xanum.uam.mx (L.G.)

[2] Facultad de Farmacia, Universidad Autónoma del Estado de Morelos (UAEM), Morelos 62209, México; cmp@uaem.mx

[3] Departamento de Ingeniería, Tecnológico de Estudios Superiores del Estado de México (TESOEM), Estado de México 56400, México; maria.lozano@tesoem.edu.mx

[4] Centro de Investigación en Química Aplicada (CIQA), CONICET, Departamento de Ingeniería Química, Facultad regional de Córdoba, Universidad Nacional Tecnológica (UTN), Maestro López esq. Cruz Roja, Córdoba 5016, Argentina; pdalmasso@frc.utn.edu.ar

[5] Instituto de Investigaciones en Físico-Química de Córdoba (INFIQC), Universidad Nacional de Córdoba (UNC), Av. Haya de la Torre, 5000., Córdoba, Argentina; grivas@fcq.unc.edu.ar

* Correspondence: elvisortiz@xanum.uam.mx (E.O.-S.)

Keywords: cholesterol oxidase; hydrogen peroxide; sensing platform; molecular recognition; in silico studies

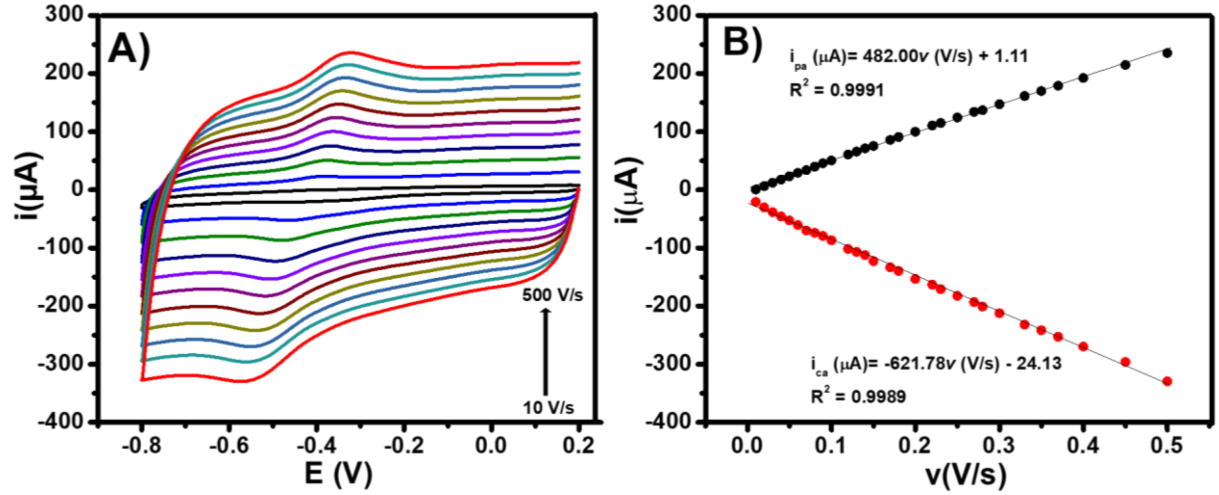

Figure S1. A) Cyclic voltammograms in PB at pH 7.4 in a potential window of -0.80 to 0.2 V, at different scan rates: 0.01, to 0.50 V/s. B). Peak current vs scan rate (*i vs v*).

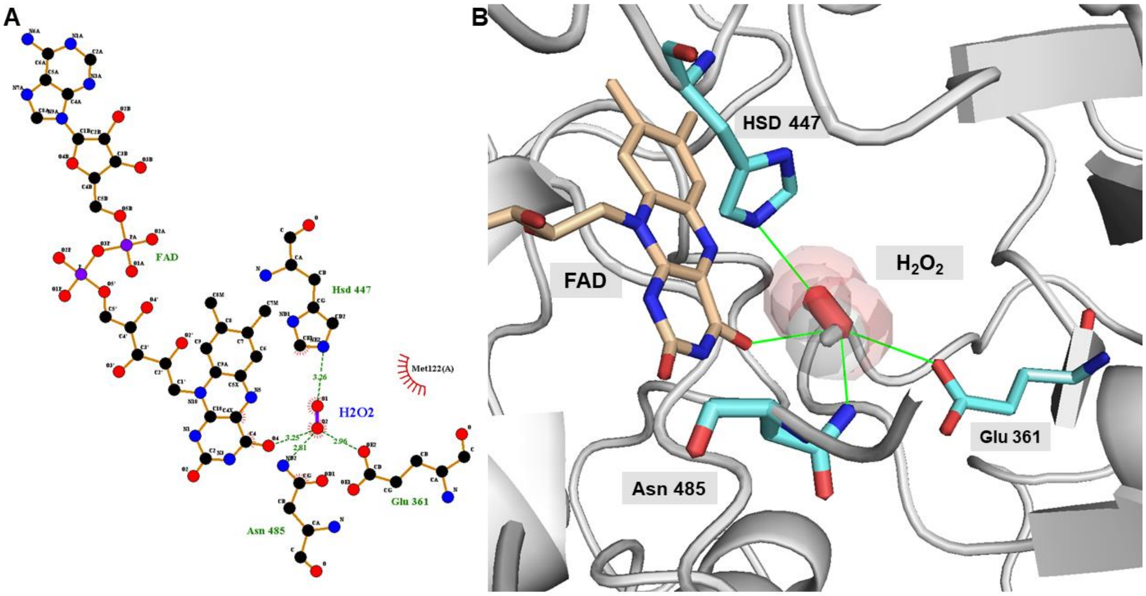

Figure S2. Schematic representation of the interaction of the FAD cofactor and H_2O_2 to cluster representative structure of ChOx: (A) Interaction diagram in 2D, showing hydrogen bonds (green color, dotted line) determined by LigPlot+ program [94]. (B) Interaction map in 3D, showing hydrogen bonds (green color line) determined by PyMOL 2.4 program [95]. HIS 447 changed to HSD 447 for the proton at Nδ on its side chain by PDB2PQR web server [96].

After performing the computational study of the molecular docking of ChOx and H_2O_2, it is corroborated that indeed there is an interaction of the hydrogen peroxide with the active center of the FAD enzyme. This allows us to affirm in more detail, that indeed the enzyme-H_2O_2.

References

[94]. Laskowski, R.A.; Swindells, M.B. LigPlot+: Multiple ligand-protein interaction diagrams for drug discovery. *J. Chem. Inf. Model.* **2011**, *51*, 2778–2786.

[95]. Schrodinger, LLC, “The PyMOL Molecular Graphics System, Version 1.3r1,” Schrodinger, LLC, New York, **2010**. http//www.pymol.org.

[96]. Jurrus, E.; Engel, D.; Star, K.; Monson, K.; Brandi, J.; Felberg, L.E.; Brookes, D.H.; Wilson, L.; Chen, J.; Liles, K.; et al. Improvements to the APBS biomolecular solvation software suite. *Protein Sci.* **2018**, *27*, 112–128. https://doi.org/10.1002/pro.3280
